# Supplementary material for: Effect of Nucleic Acid Screening Measures on COVID-19 Transmission in Cities of Different Scales and Assessment of Related Testing Resource Demands—Evidence from China
Source: Int J Environ Res Public Health. 2022 Oct 16;19(20):13343. doi: 10.3390/ijerph192013343 (PMC9602617; doi:10.3390/ijerph192013343)
Supplement: Supplementary file 1 [file ijerph-19-13343-s001.zip › ijerph-1904606-supplementary.pdf]

Supplementary Material

Table S1. Definition of COVID-19 cases. **Figure S1.** Simulated and real world values based on the SVEAI$_i$QHR model. **Table S2.** COVID-19 resource consumption proportion of confirmed patients and asymptomatic infected persons. **Table S3.** Number of Infected Persons under Scenarios 1-4 in Different Scale Cities. **Figure S2.**Change of human resource demand for laboratory testing .

After susceptible patients are infected with novel coronavirus pneumonia, they can be further divided into asymptomatic, mild, and severe patients according to the occurrence of symptoms and the severity of symptoms. The following are the definitions of asymptomatic, mild, and severe patients according to the COVID-19 diagnostic guidelines.

Table S1. Definition of COVID-19 cases

Definition of case concept	asymptomatic case	Confirmed case, non-severe	Confirmed case, severe/critical
Definition	No clinical symptoms, but the respiratory tract samples were positive for novel coronavirus[51,52]	These people have fever, general fatigue and cough, and may have mild pneumonia[53]	In addition to the symptoms of mild patients, any of the following occurs:①Shortness of breath, respiratory rate (RR) > 30 times / min; ②Oxygen saturation < 93% at rest; ③Arterial oxygen partial pressure (PaO2) / oxygen concentration (FiO2) < 300mmhg;④Pulmonary imaging showed that the lesions progressed significantly within 24–48 hours, and those with > 50% were managed as severe cases; ⑤Respiratory failure, shock and other organ failure require ICU monitoring and treatment[53]
Possible medical services	Nucleic acid testing and diagnosis, inpatient care, medical treatment	Nucleic acid testing and diagnosis, inpatient care, medical treatment	Nucleic acid testing and diagnosis, inpatient care, medical treatment, oxygen therapy, invasive mechanical ventilation, airway management, extracorporeal membrane oxygenation

As strict prevention and control measures will be taken immediately after an epidemic occurs in mainland China, so that the epidemic can be controlled in a short period of time and the epidemic time is short, it is difficult to verify the model by using the data of the fifth round of epidemic in Hong Kong. The results showed that the mean absolute percentage error (MAPE) was 1.67%, and the correlation coefficient R was 0.998. The results showed that the model fitted well, and the simulated curve was in good agreement with the epidemic data in Hong Kong.

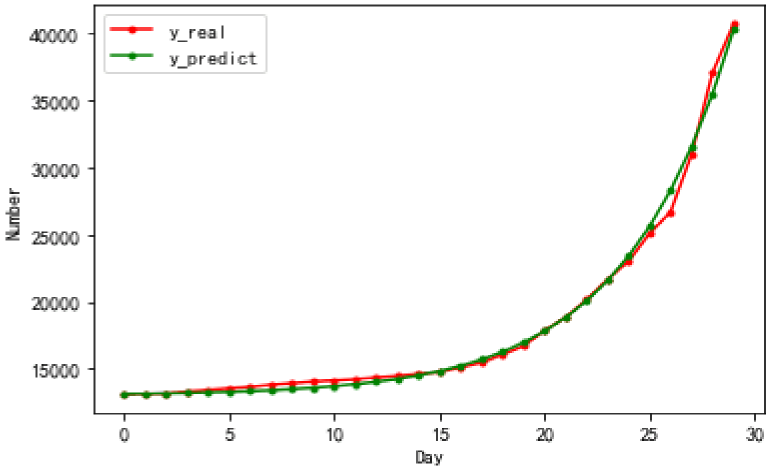

Figure S1. Simulated and Hongkong real world values based on the SVEAI_iQHR model

According to the novel coronavirus epidemic characteristics, literature reports, prevention and control guidelines, expert consultation results, and scenario assumptions, we calculated the number of laboratory testing personnel and materials-related resources. For example, according to the above models to predict in different situations is now under the circumstance of the prevention and control measures of implementing the peak number of cases and asymptomatic infections, the proportion of patients with mild and severe/critical and different scale urban population the number of clinics and determine the regular and full range of Nucleic Acid screening and detection, the rest of the indicators are specifically described in the table.

Table S2. The proportion of various resources consumed by people infected with COVID-19

Category	Meaning	Reference value	Value source
Average daily number of fever clinic visits	As the number of daily routine nucleic acid testing	Large, medium and small cities have 1786、893 and 89 people respectively	Statistical Yearbook[37]
Average daily number of new hospital admissions	As the number of daily routine nucleic acid testing	Large, medium and small cities have 5204、2602 and 260 people respectively	Statistical Yearbook[37]
Number of escorts	As the number of daily routine nucleic acid testing	Large, medium and small cities have 5204、2602 and 260 people respectively	Statistical Yearbook[37]
Close contact	Include the number of daily routine tests and the number of nucleic acid tests for all staff	Close: Infected=1:10	Guide announcement[54]
Secondary contact	Include the number of daily routine tests and the number of nucleic acid tests for all staff	Close:secondary contact=1:2	Guide announcement[54]
**Human resources**			
Sampling personnel	Information scanning is adopted to register information at sampling points	The ratio of sampling staff, service guarantee personnel and personnel to be inspected is 1:360.	Guidelines for the implementation of regional nucleic acid screening organizations[39]
Sampling service support personnel	Including sampling site information input, sample sorting and storage, and transportation personnel	The ratio of sampling staff, service guarantee personnel and personnel to be inspected is 1:3:130	Guidelines for the organization and implementation of novel coronavirus nucleic acid detection for all staff (Second Edition)[54]

Laboratory testing personnel	24 novel coronavirus nucleic acid detection personnel required for daily detection of 10,000 tubes	Sampling tube: laboratory tester = 10,000:24	Guidelines for the organization and implementation of novel coronavirus nucleic acid detection for all staff (Second Edition)[54]
Laboratory related auxiliary personnel	15 auxiliary personnel required for daily detection of 10,000 tubes	Sampling tube: laboratory assistant = 10,000:15	Guidelines for the organization and implementation of novel coronavirus nucleic acid detection for all staff (Second Edition)[54]
**Testing materials**			
Nucleic acid extraction instr ument (96 wells)	4 sets of 96 well nucleic acid extraction instruments are required for every 10,000 tubes tested	Sampling tube: nucleic acid extraction instrument (96 wells) = 10,000:4	Guide announcement[54]
Fluorescent PCR amplification instrument (96 wells)	10 fluorescent PCR amplifiers (96 wells) are required for every 10,000 tubes tested	Sampling tube: fluorescent PCR amplification instrument (96 wells) = 10,000:10	Guide announcement[54]
A2 type double biological safety cabinet	Three A2 type double person biosafety cabinets are required for every 10,000 tubes tested	Sampling pipe: A2 type double person biosafety cabinet = 10,000:3	Guide announcement[54]
Micro adjustable sampler (single channel)	4 micro adjustable sample dispensers (single channel) are required for every 10,000 tubes tested	Sampling tube: Micro adjustable sampler (single channel) = 10,000:4	Guide announcement[54]
Micro adjustable sampler (8 channels)	Three micro adjustable sample dispensers (8 channels) are	Sampling tube: Micro adjustable sampler (8 channels) = 10,000:3	Guide announcement[54]

	required for every 10,000 tubes tested		
Single tube palm centrifuge	Two single tube palm centrifuges are required for every 10,000 tubes tested	Sampling tube: single tube palm centrifuge = 10,000:2	Guide announcement[54]
Eight joint pipe	Two sets of eight joint tubes are required for every 10,000 tubes tested,	Sampling pipe: Eight joint pipe = 10,000:2	Guide announcement[54]
96 well plate centrifuge	Two 96 well plate centrifuges are required for every 10,000 tubes tested	Sampling tube: 96 well plate centrifuge = 10,000:2	Guide announcement[54]
Small vortex mixer	Two small vortex mixers are required for every 10,000 tubes tested	Sampling tube: small vortex mixer = 10,000:2	Guide announcement[54]
Multi tube vortex mixer	One multi tube vortex mixer is required for every 10,000 tubes tested	Sampling tube: multi tube vortex mixer = 10,000:1	Guide announcement[54]
Eight channel pipette	One eight channel pipette is required for every 10,000 tubes tested	Sampling tube: eight channel pipette = 10,000:1	Guide announcement[54]
Sample feeder rack	5 sample feeder racks are required for every 10,000 tubes tested	Sampling tube: eight channel pipette = 10,000:5	Guide announcement[54]
Temperature box for inactivation	Three fire-fighting temperature boxes are required for every 10,000 pipes tested	Sampling pipe: temperature box for inactivation = 10,000:3	Guide announcement[54]
Super clean workbench	One ultra clean workbench is required for every 10,000 tubes tested	Sampling pipe: super clean workbench = 10,000:1	Guide announcement[54]
-20 ℃ freezer	2 sets of - 20 ℃ freezers are required for every 10,000 tubes tested	Sampling pipe: - 20 ℃ freezer = 10,000:2	Guide announcement[54]

Movable UV lamp	8 movable UV lamps are required for every 10,000 tubes tested	Sampling tube: movable UV lamp = 10,000:8	Guide announcement[54]
Inner row autoclave	Two internal row autoclaves are required for every 10,000 tubes tested	Sampling tube: inner row autoclave = 10,000:2	Guide announcement[54]
Air disinfector	Three air disinfection machines are required for every 10,000 tubes tested	Sampling pipe: air disinfector = 10,000:3	Guide announcement[54]
**Protective materials**			
N95 and above protective mask<br>Protective clothing<br>Isolation Gown<br>Latex gloves<br>Waterproof boot cover	Disposable protective articles: sampling personnel, sampling service support personnel, laboratory testing personnel and laboratory related auxiliary personnel need protective articles	Sampling personnel: sampling service support personnel: laboratory testing personnel: laboratory related auxiliary personnel: relevant protective materials = 3:1:3:3:3	References and expert consultation[23]
Face screen or goggles	Recyclable	Disposable protective articles: face screen or goggles = 3:1	References and expert consultation[23]

The model was constructed according to the previous hypothetical scenarios, and the model was run under different city sizes to count the number of asymptomatic, confirmed, mild and severe patients and the days when the epidemic reached its peak under different scenarios.

Table S3. Number of Infected Persons under Scenarios 1-4 in Different Scale Cities

**Large city**		**Peak number of asymptomatic infections**	**Peak number of confirmed patients**	**Peak number of mild patients**	**Peak number of severe/critical patients**	**Number of days to reach the peak of the epidemic (d)**
	Scenario1	994,291	162,614	149,144	13,470	114
	Scenario2	472,547	67,293	56,975	10,318	56
	Scenario3	44	6	5	1	6
	Scenario4	65	9	8	1	17
**Medium city**						
	Scenario1	498,168	81,470	74,725	6744	110
	Scenario2	236,615	33,686	28,521	5165	54
	Scenario3	33	5	4	1	6
	Scenario4	44	6	5	1	14
**Small city**						
	Scenario1	50,347	8238	7,552	686	90
	Scenario2	23,790	3376	2,858	518	45
	Scenario3	21	5	4	1	2
	Scenario4	28	5	4	1	7

The model was constructed according to the hypothetical scenarios in the early stage, and the model was run under different city sizes to count the curves of human resources including nucleic acid sampling personnel, sampling service auxiliary personnel, laboratory testing personnel, and laboratory-related auxiliary personnel under different scenarios over time. By observing the changes in the curves, it was convenient for government departments to arrange manpower reserves.

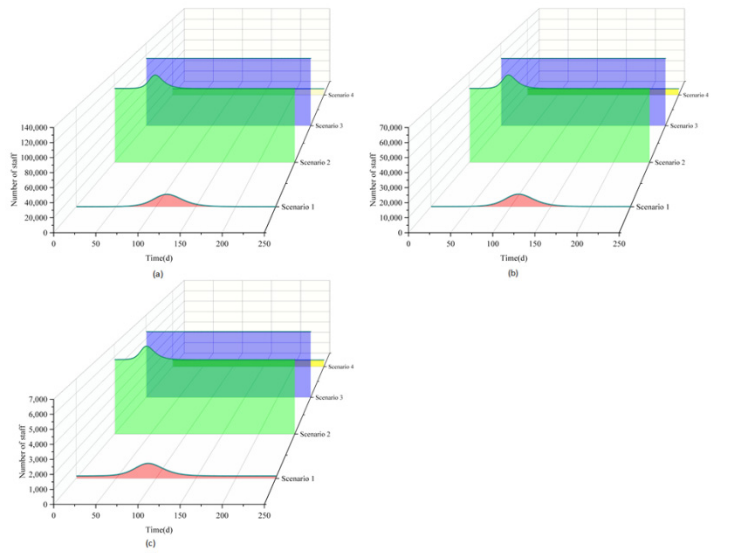

Figure S2. Human resource demand under different scale city scenarios 1-4, (a) large cities; (b) medium cities; (c) small cities
